# ENHANCING COLLABORATION FROM ADULT PROTECTIVE SERVICES TO PROVIDERS WORKING WITH MISTREATMENT VICTIMS/SURVIVORS

**DOI:** 10.1093/geroni/igad104.1570

**Published:** 2023-12-21

**Authors:** Pi-Ju Liu, Wei- Lin Xue, Zachary Hass

**Affiliations:** Purdue University, West Lafayette, Indiana, United States; Purdue University, West Lafayette, Indiana, United States; Purdue University, West Lafayette, Indiana, United States

## Abstract

Adult Protective Services (APS) is the frontline agency that receives, investigates, and provides services and/or referrals to victims/survivors of adult abuse, neglect, and exploitation. However, APS cannot resolve adult mistreatment alone, and must collaborate with healthcare, social, legal, and other community-based providers to address the needs of victims/survivors. Partnering with Montana APS and healthcare, legal, social and aging services providers, AARP, American Indian Health, local law enforcement, judge, attorneys, Medicaid Fraud Control Unit, ombudsman, and Certification and Licensing Bureaus, our goal is to examine how state agencies can work together to reduce mistreatment and systems burden. Twenty-two interviews were conducted with state agencies to inquire about collaboration challenges and strategies, what has worked well with APS, and what can be improved. Thematic analysis was used to characterize barriers and solutions for collaboration, and social network analysis was used to map out the collaboration network. We found that information sharing is a boon for collaboration among agencies using integrated information systems, but becomes a barrier when information sharing is limited by statute. State agencies also identified other agencies they have worked with to address mistreatment and reflected on challenges and strategies in working with these other professionals. Challenges for state agencies to work together center around lack of funding and service shortages, especially during the pandemic. Strategies to improve collaboration involve agency cross-training to remove silos. These interviews offer rich insight to guide practitioners across agencies to work together at the state-level to reduce mistreatment and its recurrence.

